# Sex-specific trajectories of hippocampal aging: structural changes and asymmetry across the lifespan

**DOI:** 10.1007/s00234-025-03717-8

**Published:** 2025-07-30

**Authors:** Sebastiano Vacca, Antonella Balestrieri, Carola Politi, Alessandra Serra, Luca Saba

**Affiliations:** 1 School of Medicine and Surgery, University of Cagliari, Cagliari, Italy; 2 The Feinstein Institutes for Medical Research, Northwell Health, Manhasset, NY USA; 3 Elmezzi Graduate School of Molecular Medicine, Northwell Health, Manhasset, NY USA; 4 Department of Radiology, Azienda Ospedaliero-Universitaria (A.O.U.), di Cagliari—Polo di Monserrato, Cagliari, Italy

**Keywords:** MRI, Artificial intelligence, Hippocampus, Aging

## Abstract

**Background:**

This study examines the interaction between age and sex on hippocampal volume across the lifespan, focusing on structural changes and asymmetry patterns. Extensive research has demonstrated sex differences in hippocampal structure and function, but the effects of aging on these patterns remain underexplored. Our study aims to elucidate how age-related changes in hippocampal volume differ between men and women, and their implications for cognitive and emotional processes.

**Methods:**

We performed correlational analyses of hippocampal subfield volumes, total hippocampal volume, and asymmetry indices in males and females. Age-related trends were assessed using linear mixed models (LMM), focusing on hippocampal subfields such as CA1, CA2-3, and CA4-DG. Sex-specific aging patterns were also examined using principal component analysis (PCA) to account for variance in hippocampal volume.

**Results:**

Males showed stronger asymmetry patterns, particularly between CA4-DG and CA2-3 subfields (*p* < 0.001), whereas females showed a distinct asymmetry pattern between CA4-DG and total hippocampal asymmetry (*p* < 0.001). Significant age-related hippocampal volume loss was observed in males (*p* = 0.003) but not in females (slope = 0.0026, *p* = 0.673). Sex differences were most pronounced in the 40s and 50s groups (*p* < 0.005). Additionally, males showed significant increase in CA1% volume with age (*p* = 0.038), while females did not (*p* = 0.616).

**Conclusions:**

These findings reveal distinct sex-specific trajectories of hippocampal aging, with males showing more pronounced age-related atrophy and stronger lateralization than females. The results highlight the need for sex-specific approaches in interventions aimed at mitigating hippocampal atrophy.

## Introduction

The advent of advanced neuroimaging techniques has led to a growing body of research focusing on understanding the structural changes in the brain that occur throughout the human lifespan. Among these changes, a reduction in the volume of specific brain regions, such as the hippocampus, has received particular attention due to its association with cognitive decline and age-related neurological disorders [1–3]. The hippocampus, a vital structure for memory formation and spatial navigation, is understood to experience a reduction in volume with advancing age [4, 5]. Typical hippocampal volume change across the adult lifespan is thought to exhibit a non-linear trajectory that follows a weakly declining slope until around 50 years of age when the trajectory trends downward at an accelerating rate [4–6]. This trajectory has been demonstrated by a number of comprehensive cross-sectional studies investigating age-related change in brain structure across the adult lifespan [4, 7], with middle-aged males exhibiting a greater degree of atrophy than females; however, this was not observed in older age.

As earlier, prior research has consistently demonstrated that hippocampal volume decreases with age; [8, 9] however, the extent and pattern of this decline appear to be influenced by sex. For example, some research indicates that males may experience more pronounced hippocampal atrophy as they age [10], while other studies report a more complex interaction between sex and age, with differing effects observed in various age groups [11]. Nevertheless, the evidence remains inconclusive, with some studies reporting significant sex differences in age-related hippocampal volume loss, while others find no such interaction [12, 13]. 

The discrepancies in the literature may be attributed to differences in study design, sample characteristics, and the methodologies employed to measure brain volumes. Many earlier studies utilized manual segmentation techniques to assess hippocampal volumes. While these methods have provided valuable insights, they are often limited by their reliance on large smoothing kernels and potential inaccuracies in spatial registration, which may overlook subtle sex-related differences in brain structure [14]. 

To address these limitations, our study employs the VolBrain system, a state-of-the-art automated brain MRI segmentation tool [15], to analyze hippocampal volumes in a large cohort of healthy subjects [16]. By employing this precise and objective methodology, even though trained on manual segmentations, our objective is to provide a more detailed examination of the effects of sex and age on hippocampal volumes. Specifically, this study examines how these factors interact to influence hippocampal volume across the adult lifespan, with a focus on identifying potential sex-specific patterns of hippocampal atrophy.

Gaining insight into the interplay between sex and age on hippocampal volumes is vital not only for elucidating the mechanisms underlying age-related cognitive decline but also for enhancing the precision of diagnostic and prognostic tools for neurological conditions such as Alzheimer’s disease. By elucidating the nature of these interactions, our study aims to contribute to a more nuanced understanding of brain aging and its implications for both clinical practice and cognitive health.

## Methods

### Subjects

The study utilized the publicly available IXI Dataset, comprising 662 scans from healthy, normal subjects. Data were collected from three London hospitals: Hammersmith Hospital, Guy’s Hospital, and the Institute of Psychiatry. The dataset provides comprehensive demographic and imaging data in NIFTI format. This resource was developed as part of the “IXI– Information eXtraction from Images” project (EPSRC GR/S21533/02) and is available under the Creative Commons CC BY-SA 3.0 license.

The MRI scans acquisition parameters are the following: Philips Medical Systems Intera 3T: Repetition Time = 9.60000038146972, Echo Time = 4.60269975662231, Number of Phase Encoding Steps = 208, Echo Train Length = 208, Reconstruction Diameter = 240.0, Acquisition Matrix = 208 × 208, Flip Angle = 8.0.

### Data preprocessing

**Fig. 1:**
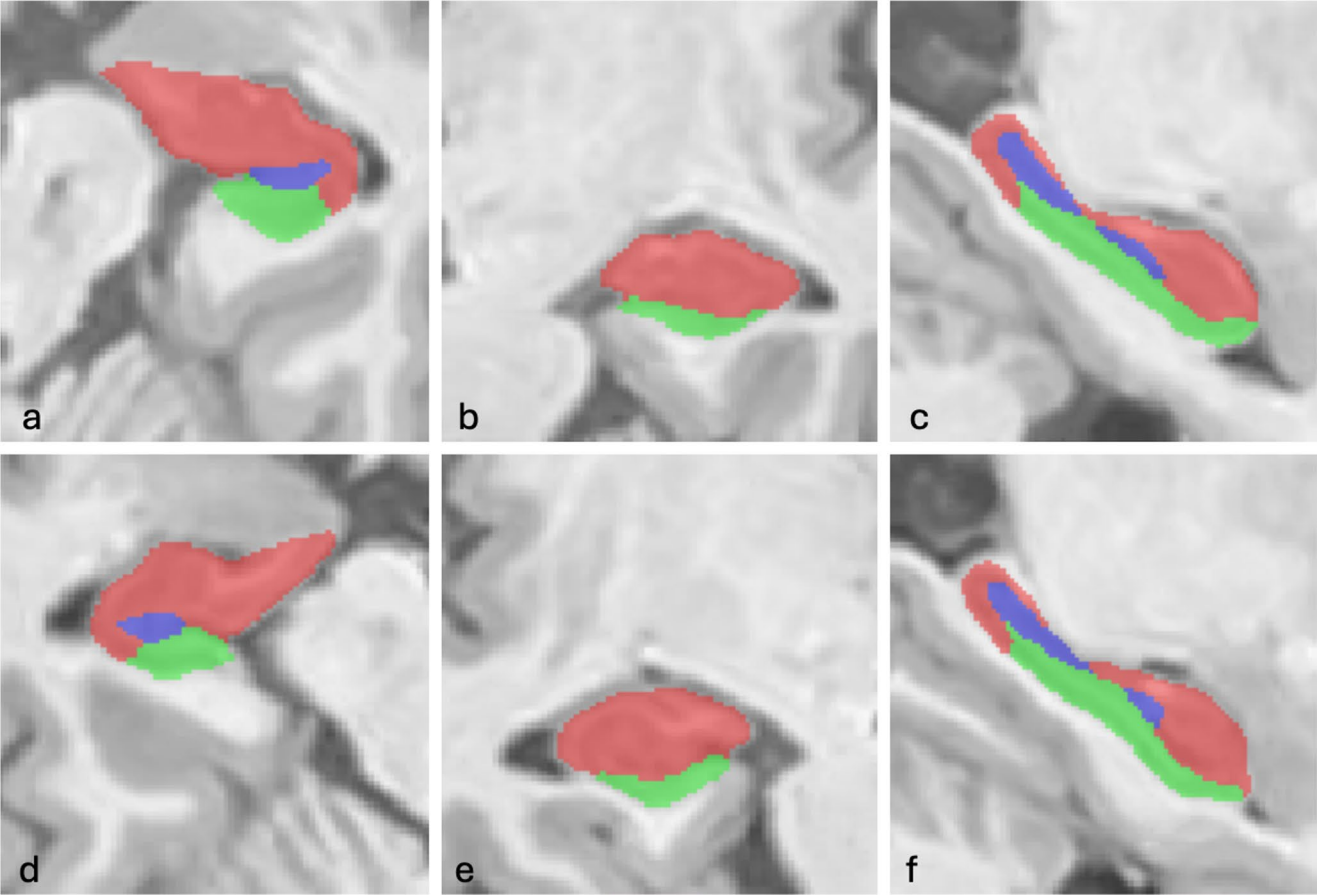
Segmentation image of the hippocampus generated by Volbrain. The upper panel (**a**, **b**, **c**) shows the right hippocampus in axial, coronal, and sagittal planes, respectively, while the lower panel (**d**, **e**, **f**) represents the left hippocampus, shown in axial, coronal, and sagittal planes, respectively

The T1-weighted images were processed by volBrain [15], an advanced pipeline that automatically provides volumetric information from brain MR images at different scales. We used HIPS on the T1-weighted images, a pipeline for automatic hippocampus subfield segmentation for monomodal (T1) or multimodal MRI data (T1 + T2); (Fig. 1), with Winterburn’s labels such as CA1, CA2-3, CA4-DG, SR-SL-SM and Subiculum. The asymmetry index is calculated as the difference between right and left thicknesses divided by their mean (in percent) [17]:

Asymmetry = (left volume - right volume)/mean (left volume, right volume) × 100%. It refers to the arithmetic mean (average) of the left and right volumes, calculated as (left volume + right volume)/2. The asymmetry index indicates the degree of left lateralization.

### Statistical analysis

All statistical analysis has been conducted on Python (version 3.10) and MATLAB (Release 2023b). We first grouped subjects by age group and sex, and then performed a Mann-Whitney test on each volumetric variable to test for significant differences between age groups and sexes. We performed a Pearson’s correlation analysis between each variable, first for both sexes and then for males and females separately. We then analyzed the slope of volume loss with age for the different hippocampal subfields.

We built a linear mixed model (LMM) that included both fixed effects (age group, sex, and their interaction) and random effects (subject-specific variations) to account for the hierarchical structure of the data. Specifically, the formula used was: {var} ~ Age_Group * Sex; with Patient_ID as the grouping variable for the random effects. The comparison age group was 40–49. A multivariate analysis of covariance (MANCOVA) was also performed to assess the effect of age group, sex, and their interaction on the dependent variables. Finally, a Principal Component Analysis (PCA) was performed to check the amount of variance explained by each hippocampal variable.

## Results

### Demographics

Of the original 662 patients, 362 were excluded due to processing errors related to image quality. The demographic data for the remaining 300 patients are included in Table 1. The population comprised 114 males and the average age was 47.9 years. The age groups were divided by decade.

**Table 1:** Demographic information of the study participants

Metric	Gender	Mean	Range	Standard Deviation
Gender Count	Males	114 (38%)	N/A	N/A
	Females	186 (62%)	N/A	N/A
Age (years)	Males	47.67	19.9–84.2	16.8
	Females	50.81	20.1–86.3	16.6
Height (cm)	Males	168.87	113.6–210	23.7
	Females	153.66	110–197.2	21.7
Weight (kg)	Males	75.62	49.3–127	19.2
	Females	65.8	47–102.2	18.2

### Volumetric differences

The Mann-Whitney test, conducted on sex differences at every age group, yielded various significant results for each hippocampal variable. For the Hippocampal total volume, we found highly significant differences between sexes in the 40–49 and 50–59 age groups (p-value < 0.005), as well as significant ones in the 60–69 and 80–89 age groups (*p* < 0.05). This is also reflected in the right and left hippocampal volumes and the CA1 total volume, as for the same age groups we found a significant difference (p-value < 0.05). Regarding the CA2-3 total volume, the difference was notable only in the 40–49 and 50–59 age groups (p-value < 0.05), while for the CA4-DG total volume we found a significant difference (p-value < 0.05) in every group besides the 90–99 and 60–69 ones.

**Fig. 2:**
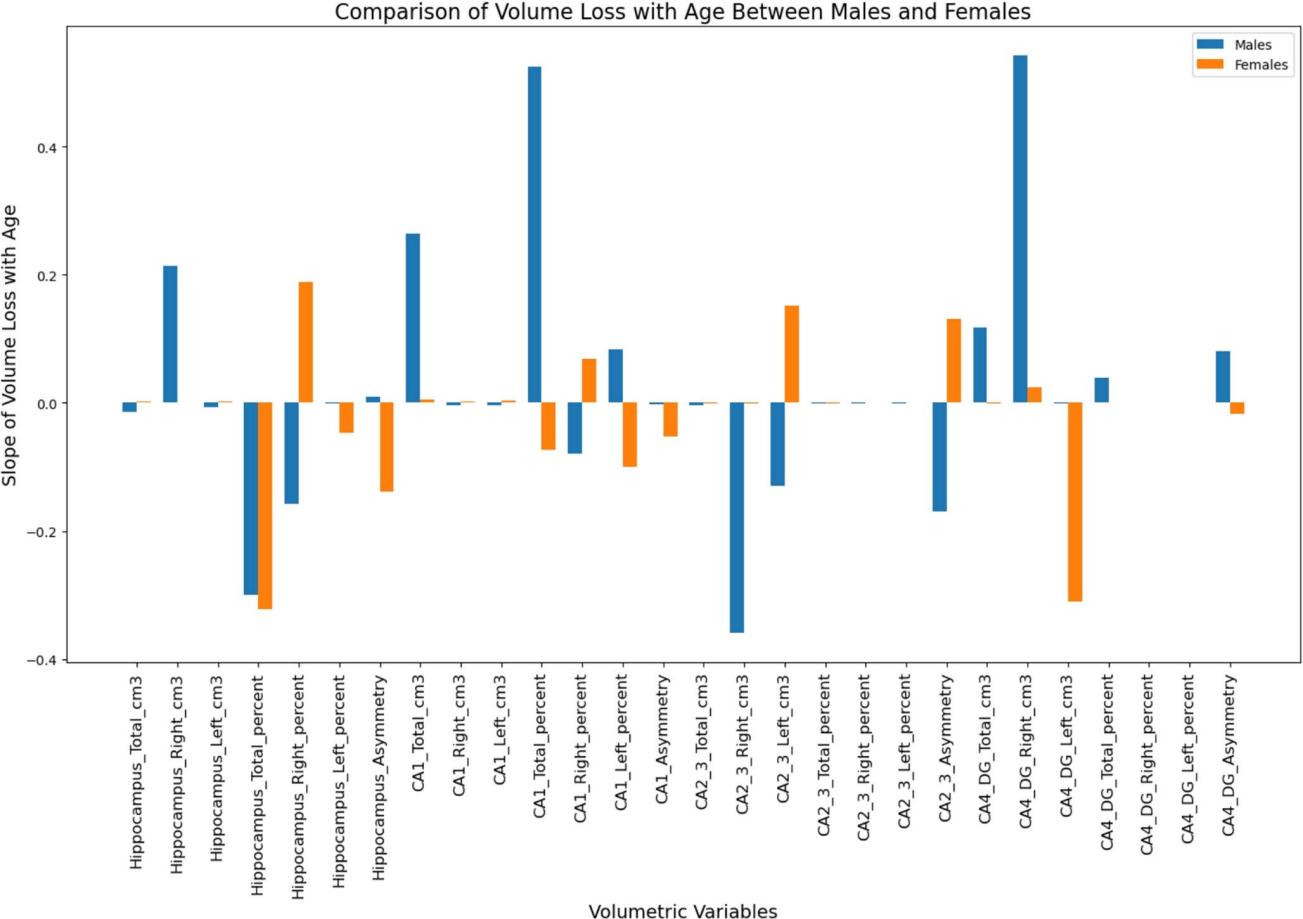
Slope of volume loss, between males (blue) and females (orange), with age for various hippocampal subfields. Positive slopes indicate volume increases with age, while negative slopes indicate volume loss

Males show a significant decline in hippocampal total volume with age (slope = −0.0137, *p* = 0.003), while females do not exhibit a significant change (slope = 0.0026, *p* = 0.673). (Fig. 2) A notable difference is observed in the CA1 total volume percent between sexes. Males have a positive slope (0.5243, *p* = 0.038), indicating an increase, whereas females have a slight negative slope (−0.0730, *p* = 0.616). This difference is statistically significant, as shown by the U-test results (u-statistic = 0.87, p-value = 0.021), indicating a greater increase in CA1 volume percent with age in males compared to females. Both males and females experience a decline in CA2-3 total volume with age, but the rate of decline is more pronounced in males (slope = −0.0033, *p* < 0.005) compared to females (slope = −0.0014, *p* = 0.106). In terms of asymmetry, males show an increase in CA4-DG asymmetry (slope = 0.080, *p* = 0.134), while females show a decrease (−0.018, *p* = 0.672).

### Correlation analysis

**Fig. 3:**
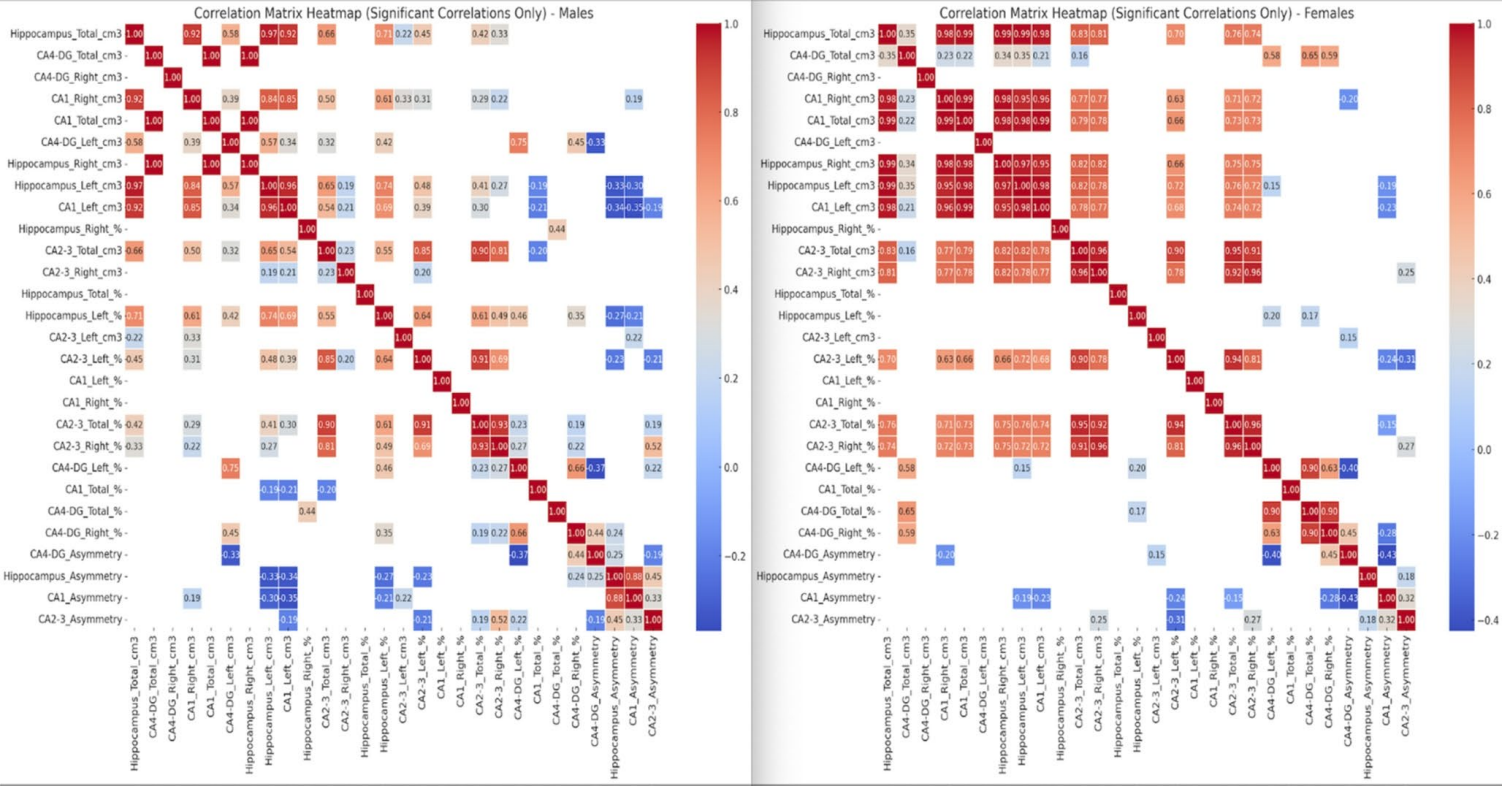
Correlation Heatmap of significant correlations (*p* < 0.05) only, between hippocampal variables. Left males, right females

Correlation analysis for males revealed several significant relationships between hippocampal subfield volumes, total hippocampal volume, and asymmetry indices. In particular, total hippocampal volume showed strong positive correlations with total CA4-DG volume (*r* = 1.00), total CA1 volume (*r* = 0.97), and left hippocampal volume (*r* = 0.97). Regarding asymmetry, CA4-DG asymmetry was positively correlated with CA2-3 asymmetry (*r* = 0.80) and hippocampal asymmetry (*r* = 0.75). However, CA2-3 asymmetry showed a weak correlation with total hippocampal volume (*r* = 0.22) (Fig. 3).

Similar significant correlations were observed in females, with total hippocampal volume strongly positively correlated with total CA4-DG volume (*r* = 0.99), total CA1 volume (*r* = 0.98), and right hippocampal volume (*r* = 0.98). Notably, the correlation between CA2-3 total volume and hippocampal total volume was slightly stronger (*r* = 0.83) compared to males (0.66). Asymmetry indices in females also showed significant correlations, with CA4-DG asymmetry positively correlated with hippocampal asymmetry (*r* = 0.90) and CA2-3 asymmetry (*r* = 0.63). Interestingly, hippocampal asymmetry showed a weak correlation with left CA2-3 volume percent (*r* = 0.15) (Fig. 3).

### Linear-mixed-model

Significant interactions between sex and age groups were found in hippocampal asymmetry, with males generally exhibiting higher asymmetry than females. The difference in asymmetry was particularly pronounced in the 50–59 (β = −4.256, *p* = 0.030), 60–69 (β = −5.860, *p* = 0.022), and 80–89 (β = −5.602, *p* = 0.020) age groups. Additionally, significant sex differences were observed in total hippocampal volume, with the 70–79 age group showing a marked difference (β = 1.102, *p* = 0.009). For the CA1 region, significant interactions between sex and age were noted, particularly in the 60–69 (β = −255.467, *p* = 0.002) and 70–79 (β = 0.551, *p* = 0.001) age groups, indicating substantial differences in volume between males and females. Other significant findings include sex-specific differences in CA4-DG total volume in the 60–69 age group (β = −112.499, *p* < 0.001) and in CA4-DG right volume in the 80–89 age group (β = −51.477, *p* = 0.022).

### MANCOVA

The MANCOVA analysis revealed highly significant effects for the intercept across all multivariate test statistics, including Wilks’ lambda (Λ = 0.006, *p* < 0.001) and Roy’s greatest root (Θ = 161.088, *p* < 0.00). Age Group also demonstrated significant effects, particularly with Wilks’ lambda (Λ = 0.454), *p* = 0.046) and Hotelling-Lawley trace (T = 0.862, *p* = 0.035). Sex had a significant multivariate effect across all tests, with Wilks’ lambda (Λ = 0.795, *p* < 0.001) and Roy’s greatest root (Θ = 0.257, *p* < 0.001). The interaction between Sex and Age Group did not reach significance on most tests, but Roy’s greatest root (Θ = 0.215, *p* = 0.001) suggested a notable interaction.

### PCA

**Fig. 4:**
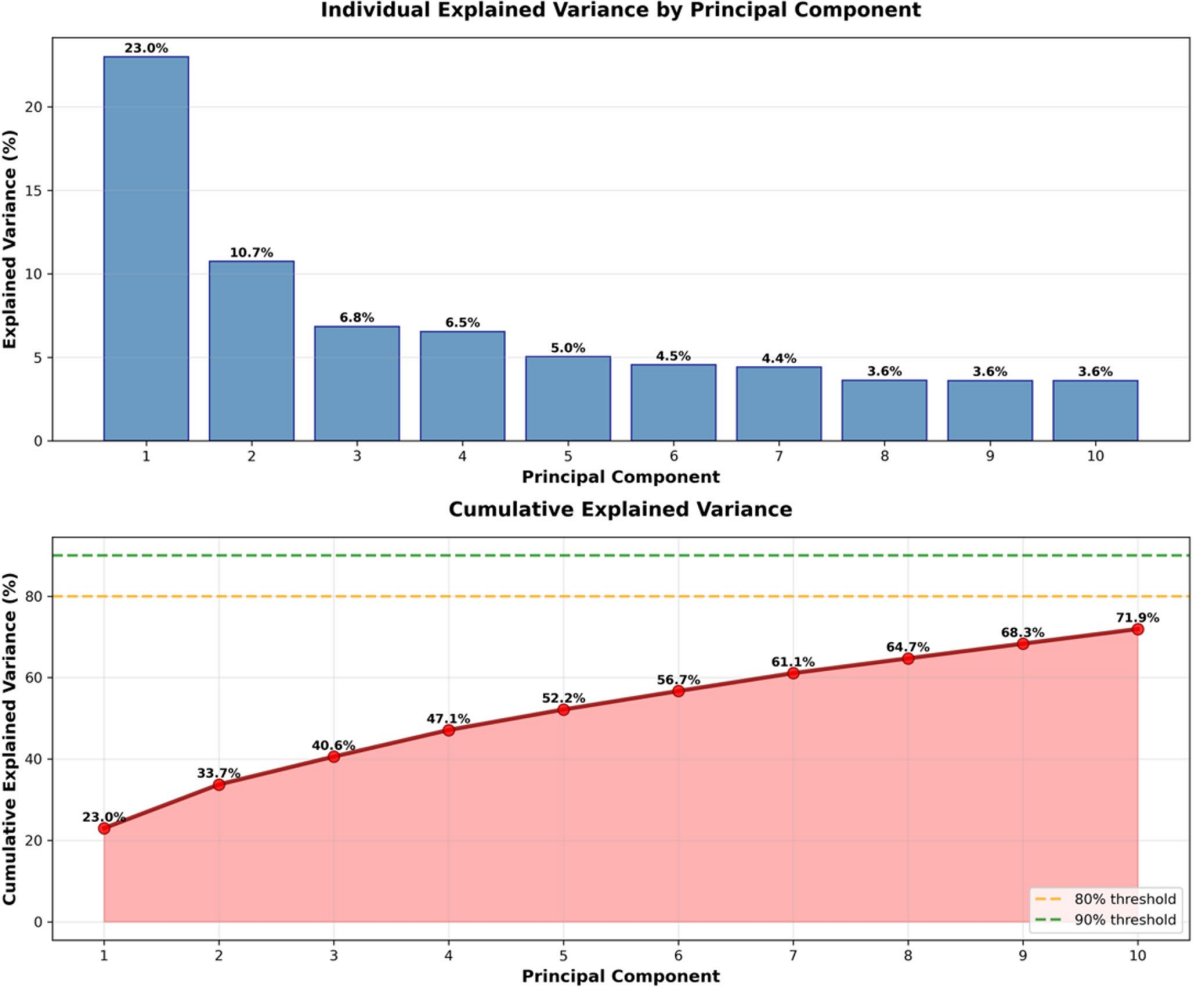
PCA of hippocampal variables, with the first five principal components (PC1 to PC5) cumulatively explain 52.2% of the variance

**Fig. 5:**
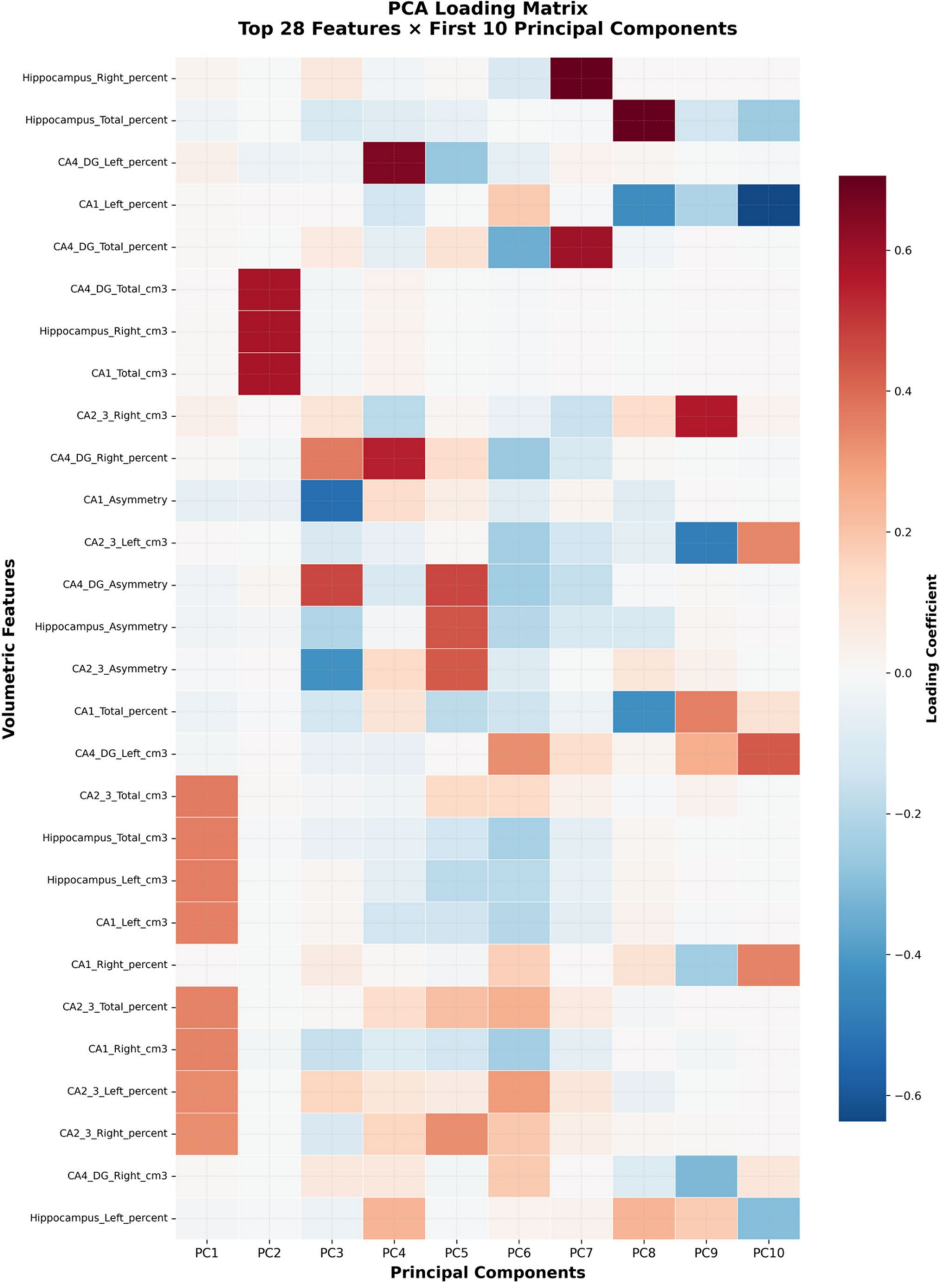
Resulting loading matrix from the PCA for the first 10 PCs (71.9% of the explained variance)

The PCA analysis revealed that the first principal component (PC1), which accounts for approximately 20% of the total variance, shows that age group is a significant factor (F = 2.588, *p* = 0.013), while sex is not (F = 0.612, *p* = 0.434). The first five principal components (PC1 to PC5) cumulatively explain around 50% of the variance. We then conducted an ANOVA to check whether age and sex significantly affected the PCs. Notably, PC4 shows a highly significant effect of sex (F = 17.158, *p* < 0.005), and PC5 also shows a significant effect of sex (F = 6.846, *p* = 0.009), while age is not significant for these components (Figs. 4 and 5).

## Discussion

Our study examines the interaction between age and sex in relation to hippocampal volume across different life stages, emphasizing the complexity of structural changes in the hippocampus and identifying notable sex-specific patterns associated with aging. Extensive research has examined structural and functional changes in the hippocampus across the lifespan, with a particular focus on sex differences, age-related decline, and the impact of genetic risk factors on hippocampal subfields.

Sex differences in hippocampal function and plasticity are evident in both rodents and humans, with women more susceptible to greater cognitive decline in diseases characterized by hippocampal dysfunction, such as Alzheimer’s disease and depression [18–21]. Notably, sex differences also influence neurogenesis in the hippocampus, with different levels of new neuron activation in response to cognitive tasks, highlighting the need to consider sex as a critical factor in hippocampal research [22, 23]. Furthermore, hippocampus contains sex hormone receptors such as androgen receptors (AR), and estrogen receptors (ER)- α, β and G-coupled protein receptor (GPER), that might drive some of the between sexes differences [24–26]. 

Age-related changes in brain volume vary by region, sex, and genetic background, with the hippocampus and subcortical structures showing different trajectories. Across studies, the hippocampus consistently shows age-related decline, though the pattern varies by subfield and sex. CA1-2 volumes tend to decrease linearly with age, while CA3-DG and the entorhinal cortex decline earlier in life and stabilize later, and the subiculum appears more resistant to age-related change [27]. In regard to pace of volume reductions, men show steeper hippocampal volume decline in midlife compared to women, with their volume loss following a nonlinear trajectory. In contrast, women exhibit a more gradual, linear decline. Hippocampal asymmetry also shifts with age in men but remains stable in women: these findings, taken together, suggest sex-specific structural aging. Furthermore, genetic factors further modulate these patterns: female ApoE e4/e4 carriers experience pronounced volume loss in hippocampal subfields after age 65, particularly in the presubiculum, subiculum head, and CA1 and CA3 regions [28].

Besides the hippocampal cortex, subcortical regions such as the putamen, pallidum, and thalamus also undergo volume reductions with age, with men again showing a faster rate of decline than women. In the thalamus, this decline follows a quadratic pattern in males, accelerating with age, while females follow a more stable trajectory [29]. Beyond these volumetric changes, certain hippocampal subfields like the parasubiculum and fimbria are consistently larger in males, even after accounting for total brain volume, though other regions do not show consistent sex differences [30].

Most of the mentioned studies are cross-sectional, but longitudinal data support these findings, indicating that hippocampal volume loss accelerates with age, more so in older adults than in midlife, and that individual variability in aging trajectories is substantial [9]. While some individuals show clear volume loss, a significant portion of older adults maintain stable hippocampal structure over time, pointing to potential resilience mechanisms.

Pearson correlation analysis revealed distinct patterns of associations between hippocampal subfield volumes, total hippocampal volume, and asymmetry indices in males and females. These findings highlight potential sex differences in hippocampal structure, which may have implications for understanding sex differences in vulnerability to neurological and psychiatric disorders. Correlation analysis revealed that males exhibited stronger and more coordinated asymmetry patterns between hippocampal subfields, particularly between CA4-DG and CA2-3 asymmetry (*r* = 0.80, *p* < 0.001), compared to females. In contrast, females showed a distinct asymmetry pattern in which CA4-DG asymmetry was more closely linked to overall hippocampal asymmetry (*r* = 0.90, *p* < 0.001), suggesting potential sex-specific mechanisms influencing hippocampal lateralization.

The observed sex differences in correlation patterns suggest that males and females may follow different developmental trajectories in hippocampal structural organization. In particular, the stronger correlations between subfield asymmetries in males may indicate a more pronounced or coordinated lateralization process in this group. These findings are consistent with previous research suggesting sex differences in hippocampal function and vulnerability to stress and neurodegeneration, possibly driven by hormonal influences [27, 28, 30]. Further research is warranted to explore the underlying mechanisms of these sex differences and their implications for hippocampal-related cognitive and emotional processes.

Our analysis reveals a significant age-related decline in total hippocampal volume in males (slope = −0.0137, *p* = 0.003), while females do not show a significant change with age (slope = 0.0026, *p* = 0.673). This suggests that aging has a greater impact on hippocampal structure in males, possibly reflecting an accelerated neurodegenerative process. The lack of significant decline in women may indicate the presence of protective factors that buffer against age-related atrophy.

Sex differences in hippocampal volume were most pronounced during midlife, particularly between the ages of 40 and 59, when men consistently showed larger hippocampal and CA1 volumes than women. This pattern was also evident, though to a lesser extent, in older age groups, suggesting that midlife may be a key window for the emergence of sex-related structural divergence in the hippocampus.

In the CA1 subregion, men showed an age-related increase in relative volume, while women showed a slight decline. This contrast points to fundamentally different aging trajectories between the sexes in this region, which is known to be vulnerable to neurodegeneration [27, 28]. Similarly, CA2-3 volume declined more steeply in men, further pointing to the hypothesis that male hippocampal subfields may be more sensitive to age-related atrophy.

Hippocampal asymmetry also changed differently with age in men and women. Men exhibited greater asymmetry in later decades, which may reflect lateralized atrophy or compensatory structural adaptations not seen in women. These findings support the broader notion that brain aging follows sex-specific paths, and not only in overall volume but in the pattern and pace of change across hippocampal subfields.

PCA results provided further evidence of sex differences in hippocampal volumes. The first principal component (PC1), accounting for approximately 20% of the total variance, was significantly associated with age group (*p* = 0.013) but not with sex (*p* = 0.434). However, PC4 and PC5 showed highly significant effects of sex (PC4: *p* < 0.005; PC5: *p* = 0.009), highlighting the importance of considering both sex and age when studying hippocampal structure.

This study has several limitations: first, the cross-sectional nature of our analysis might be a bias factor, even though conducting longitudinal studies across the lifespan is challenging. Moreover, our cohort comprised of almost exclusively subjects over 40 years old, so other studies with more age groups could help in confirming our results.

These findings have important implications for understanding sex-specific trajectories of hippocampal aging. The distinct patterns observed between males and females suggest that interventions aimed at mitigating age-related hippocampal atrophy may need to be tailored by sex. The significant sex differences in hippocampal asymmetry, as well as in specific subregions such as CA1 and CA2-3, suggest potential mechanisms that could be targeted in future research to better understand the biological underpinnings of cognitive aging. Longitudinal studies incorporating sex hormone levels and stress responses may provide further insight into the factors driving these sex-specific patterns.

## Abbreviations

CA: Cornu Ammonis
DG: Dentate Gyrus
LLM: Linear-Mixed-Model
MRI: Magnetic Resonance Imaging
PCA: Principal Component Analysis
SR-SL-SM: Stratum Radiatum/Stratum Lacunosum/Stratum Moleculare
